# Intranasal Inoculation of White-Tailed Deer (*Odocoileus virginianus*) with Lyophilized Chronic Wasting Disease Prion Particulate Complexed to Montmorillonite Clay

**DOI:** 10.1371/journal.pone.0062455

**Published:** 2013-05-09

**Authors:** Tracy A. Nichols, Terry R. Spraker, Tara D. Rigg, Crystal Meyerett-Reid, Clare Hoover, Brady Michel, Jifeng Bian, Edward Hoover, Thomas Gidlewski, Aru Balachandran, Katherine O'Rourke, Glenn C. Telling, Richard Bowen, Mark D. Zabel, Kurt C. VerCauteren

**Affiliations:** 1 National Wildlife Research Center, US Department of Agriculture, Animal and Plant Health Inspection Service, Wildlife Services, Fort Collins, Colorado, United States of America; 2 Colorado State University Diagnostic Laboratory, Fort Collins, Colorado, United States of America; 3 Prion Research Center and the Department of Microbiology, Immunology and Pathology, College of Veterinary Medicine and Biomedical Sciences, Colorado State University Prion Research Center, Fort Collins, Colorado, United States of America; 4 Department of Biomedical Sciences, College of Veterinary Medicine and Biomedical Sciences, Colorado State University, Fort Collins, Colorado, United States of America; 5 National and OIE Reference Laboratory for scrapie and chronic wasting disease, Canadian Food Inspection Agency, Ottawa, Ontario, Canada; 6 U. S. Department of Agriculture, Agricultural Research Service, Pullman, Washington, United States of America; Creighton University, United States of America

## Abstract

Chronic wasting disease (CWD), the only known prion disease endemic in wildlife, is a persistent problem in both wild and captive North American cervid populations. This disease continues to spread and cases are found in new areas each year. Indirect transmission can occur via the environment and is thought to occur by the oral and/or intranasal route. Oral transmission has been experimentally demonstrated and although intranasal transmission has been postulated, it has not been tested in a natural host until recently. Prions have been shown to adsorb strongly to clay particles and upon oral inoculation the prion/clay combination exhibits increased infectivity in rodent models. Deer and elk undoubtedly and chronically inhale dust particles routinely while living in the landscape while foraging and rutting. We therefore hypothesized that dust represents a viable vehicle for intranasal CWD prion exposure. To test this hypothesis, CWD-positive brain homogenate was mixed with montmorillonite clay (Mte), lyophilized, pulverized and inoculated intranasally into white-tailed deer once a week for 6 weeks. Deer were euthanized at 95, 105, 120 and 175 days post final inoculation and tissues examined for CWD-associated prion proteins by immunohistochemistry. Our results demonstrate that CWD can be efficiently transmitted utilizing Mte particles as a prion carrier and intranasal exposure.

## Introduction

Chronic wasting disease (CWD) is a naturally occurring transmissible spongiform encephalopathy (TSE) of deer, elk and moose that affects captive as well as wild populations. Currently, 15 states, 2 Canadian provinces and South Korea have reported cases of CWD [1] with more being reported each year. Like all prion diseases, conversion of a normal, host cellular prion protein (PrP^C^) to a pathologic, misfolded form (PrP^CWD^) causes CWD. This conversion process, according to the prion hypothesis, allows aberrantly folded prions to replicate without a genome [2]. Death inevitably occurs after onset of clinical disease. Direct animal-to-animal [3] and indirect environmental [4] CWD transmission have been shown to occur. Animal studies have demonstrated that CWD-positive deer can disseminate CWD prions into the environment via urine, feces and saliva [5]–[7]. Although the concentration of prions in urine and feces is low, the total fecal and urine output is high, with each deer depositing approximately 119 kg dry weight of feces/deer [8] and roughly 1000 L of urine per deer into the environment annually. Elk excretion of prion is likely greater as they are much larger animals. This ongoing, progressive environmental contamination may be substantial in areas with large cervid populations and a high incidence of CWD. Once in the environment, prions can persist and transmit disease years after removal of infected animals and indirect environmental transmission in deer has been documented [4], [9], [10]. After contaminated urine, feces or saliva have been deposited in the environment on forage and soil, they become available for ingestion and inhalation. Soil particles, particularly clay, tightly adsorb prions [9], [11], [12] and soil is inadvertently ingested during foraging with deer consuming an average of 16 g of soil per day [13]. Soil is also intentionally ingested at mineral licks, which are utilized by deer, elk and moose (our unpublished observations). Oral delivery of CWD-positive brain material into deer in the laboratory resulted in effective transmission of the disease [14], [15] and soil-bound prions enhanced oral transmission in hamsters [16]. In addition to oral ingestion of soil, deer and elk also stir up and inhale dust during foraging, running and rutting behaviors such as advertising, territorial, and hierarchical displays [17], [18]. Male white-tailed deer mark territory by scraping patches of earth and urinating on them. Other male and female deer then encounter the scrape, smell the soil and perform the same marking behavior [17]. Elk engage in several behaviors that can aerosolize dry soil particles: dominance displays such as thrashing the ground with their antlers as a visual performance for challenging bulls and advertising displays for females such as and pawing and scraping the ground with their antlers [18]. We propose that inhalation of CWD-contaminated dust particles during these behaviors is a likely route of exposure.

Intranasal (IN) infection has been validated in rodent models by placing liquid inoculum into or in front of the nasal cavity [19]–[21]. Kincaid and Bartz discovered that although IN inoculated hamsters had incubation times equivalent to orally inoculated hamsters, the IN route was 10–100 times more efficient at transmitting the disease [21]. This suggests that the IN route may be an efficient route of transmission in natural situations. A recent study utilizing aerosolized liquid inoculum demonstrated that CWD can be transmitted via the nose in white-tailed deer [22]. In the current study, CWD-positive brain homogenate was mixed with montmorillonite clay (Mte), lyophilized, pulverized, and atomized into the nasal cavity of white-tailed deer once a week for six weeks, modeling a chronic environmental exposure. The deer were euthanized at four time points and tissues were examined by immunohistochemistry. We found that lyophilized CWD prion particulate complexed to Mte was efficiently transmitted IN to deer and PrP genotype influenced lymphatic distribution and density.

## Materials and Methods

### Animals and husbandry

All procedures involving animals were performed to minimize suffering and were approved by the Institutional Animal Care and Use Committee at Colorado State University in accordance with the USDA Animal Welfare Act Regulation. CFR, title 9, chapter 1, subchapter A, parts 1–4.

Fifteen white-tailed deer (*Odocoileus virginianus*) fawns of mixed sex and PRNP genotype (codon 96) were purchased from a CWD-free private deer facility (Northwoods White-tails, Missouri, US) in June 2011 and transported to the USDA National Wildlife Research Center (NWRC) in Fort Collins, CO. At the time of transport fawns ranged from two to fourteen days of age. Fawns were bottle-fed and were weaned by 16 weeks. One fawn was euthanized at eight weeks of age due to an encephalitis of unknown origin.

At twelve weeks of age 12 deer were transported to a Colorado State University (CSU) BSL-2 facility for CWD-inoculation. The remaining two deer served as controls and remained at the USDA National Wildlife Research Center (NWRC) facility.

### *PRNP* Genotyping

Blood was drawn from fawns upon arrival and sent to the USDA Agricultural Research laboratory in Pullman, WA for genetic analysis of the open reading frame of *PRNP*. All animals were homozygous for the wild type amino acid at residue 95-(glutamine Q). There were three genotypes present for codon 96: 5 were homozygous for the wild type allele encoding glycine (GG), four were heterozygous for G and the alternative allele encoding serine (GS) and five were homozygous SS. To assess any effects of genotype on study results, deer from each genetic group were distributed as evenly as possible among the euthanasia time point groups.

### Inoculum preparation

**Figure 1:**
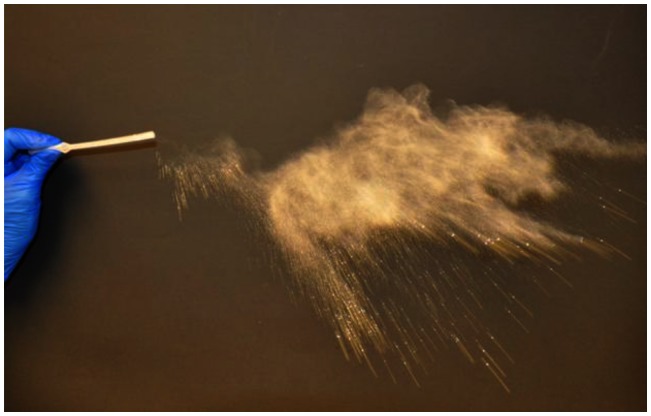
Expulsion of the lypholized CWD/Mte inoculum from a transfer pipette illustrating the ultrafine texture of the inoculum.

A 20% homogenate of white-tailed deer brain from experimentally inoculated, CWD-terminal or CWD-negative deer was generated in 0.5× phosphate buffered saline (PBS) and glass beads utilizing a Blue Bullet homogenizer (Next Advance, Averill Park, NY) as previously described [23]. To verify the negative status of the brain homogenate from the control animal, six rounds of serial protein misfolding amplification (PMCA) were conducted and samples visualized by western blot as previously described [23]. CWD status of the positive brain homogenate was verified by western blot. Individual inoculums were prepared by thoroughly mixing 1 ml of the 20% brain homogenate (200 mg total brain) and 250 mg of Mte clay (Sigma, St. Louis, MO) in a plastic weigh boat. Brain/clay slurry was lyophilized at room temperature for 24 hours under a chemical fume hood. A razor blade was used to scrape and grind the dried mixture back into a fine powder (Fig. 1). The resulting powdered inoculum was drawn into a blunt plastic transfer pipette, sealed with parafilm, and kept at −80°C until needed. To determine the size of the Mte clay particles, a sample was analyzed by the Colorado State University Soil laboratory (Fort Collins, CO) utilizing the pipette method [24].

### Inoculum titer

We quantified the infectivity of our CWD+ inoculum with the cervid prion cell assay (CPCA) as described previously [25]. Briefly, a 10% brain homogenate was made from a 20% inoculum sample with cold PBS lacking Mg^2+^ and Ca^2+^. Homogenate was passed through 18-, 23-, 26- and 28-gauge needles 15 times each, and stored at −80°C until used. A rabbit kidney epithelial (RK13) cell line (ATCC, Manassas VA) engineered to express deer prion protein, referred to as Deer5E9-S1, which is highly sensitive to the infection with deer CWD prions (manuscript in preparation) was utilized in this assay Twenty thousand cells per well were plated in 96-well plates. One day later, cells were exposed to three-fold serial dilutions of deer CWD+ brain homogenate prepared in cell culture medium, ranging from 10^−2^ to 10^−5^, in a volume of 100 µl per well. Cells that were not exposed to CWD prions served as a background control.

Cells were passaged three times at four day intervals at 1∶4 and 1∶7 split ratios. When cells reached confluence at the third passage, 20,000 cells per well were filtered onto Multiscreen IP 96-well 0.45-µm filter plates (Elispot plates, Millipore, Billerica, MA). Plates were dried at 50°C and cells were digested for 90 min at 37°C in 60 µl of lysis buffer containing 5 µg/ml proteinase K (PK) then terminated with phenylmethanesulfonylfluoride (PMSF) (2 mM). To expose the epitope of PrP27-30, cells were incubated in 120 µl 3 M guanidinium thiocyanate in 10 mM Tris-HCl (pH 8.0) for 10 min at room temperature then rinsed four times with 160 µl PBS. For immunodetection, wells were filled with 120 µl of filtered 5% superblock (Pierce, Rockford, IL) and incubated for one hr at room temperature. The solution was removed by vacuum, and wells were incubated with 60 µl of 6H4 mAb, diluted 1: 5000 in TBST for one hr at RT or overnight at 4°C. Wells were rinsed four times with 160 µl of TBST then incubated with 60 µl AP-α-Mouse IgG (Southern Biotechnology Associates, Birmingham, AL), diluted 1: 5000 in TBST, after one hr at RT, the wells were rinsed four times with 160 µl TBST, followed by a final wash with PBS. Plates were allowed to dry completely. Visualization was done by adding 60 µl of AP conjugate substrate kit (Bio-Rad, Hercules, CA) at RT and rinsing twice with 160 µl water and allowed to completely dry. Images were scanned with a ImmunoSpot S6-V analyzer (Cellular Technology Ltd, Shaker Heights, OH), and spot numbers were determined using ImmunoSpot5 software (Cellular Technology Ltd, Shaker Heights, OH). Statistical analyses were performed using GraphPad Prism 5.0 for Mac OS X software. Prion titer was calculated in spot-forming CPCA units per gram of wet brain homogenate.

### Inoculations

The deer were bottle-fed and hand-raised so they were easily manually restrained for the intranasal inoculations. The plastic transfer pipette containing the powdered inoculum was warmed to room temperature, inserted approximately 3 cm into the left nostril and inoculum puffed into the nasal cavity once a week for six weeks, exposing each deer to a total of 1.2 g (wet weight) of brain. Two mock-infected control deer were inoculated in the same fashion using CWD-negative brain/clay inoculum. Three deer were euthanized at each of three time points after the final inoculation: 95, 105, 120 days post inoculation (DPI); and two deer at 175 DPI. Because of the presence of genetic variation at codon 96, a GG, GS and SS deer was placed in each of the four time groups with the exception of the 105 DPI group, which contained one GG and two SS genotypes. The control deer were composed of one GG and one GS.

### Inoculum tracking

Two tracking methods were employed to determine the distribution of the CWD inoculum within nasal the cavity, both grossly and microscopically, after inoculation. To grossly determine the distribution of the clay inoculum, 500 µl of green fluorescent dye (GFD, Wizard Tattoo Ink, Chicago, IL) and 500 µl of deionized water were added to 250 mg of Mte clay and mixed thoroughly. The mixture was prepared and inoculated IN as described for brain homogenates. The ink was visible with both ultraviolet and visible light. One control deer was manually restrained and inoculated IN with GFD/Mte into the left nostril. Forty-five minutes later the deer was euthanized and necropsy conducted.

To track the uptake of the CWD/Mte material within the nasal mucosa, a lyophilized, fluorescently-labeled CWD prion was strategy employed. Purified deer CWD prion rods were isolated as previously described [26] and conjugated to a Dylight 650 fluorescent tag using a Dylight antibody labeling kit (Thermo Scientific Pierce, Waltham, NJ). Ten µl aliquots were each mixed with 50 µl of deionized H_2_O then floated in the water bath of a 3000MP sonicator (Misonix, Farmingdale, NY) at 37°C and sonicated for 30 sec at 70% maximum power. Sonicated, fluorescent prions were mixed with an additional 450 µl of DI H_2_O then with 250 mg of Mte clay in a weigh boat and lyophilized. Prion/Mte dust was prepared and inoculated as the brain inocula described above. The two deer from the final 175 DPI euthanasia time point were manually restrained and given the prion/Mte inoculum in the left nostril at either 60 or 45 min prior to euthanasia.

Deer were sedated with Xylazine (Lloyd Laboratories, Shenandoah, IA) then euthanized via intravenous injection of Beuthanasia-D (Schering-Plough Animal Health Corp., Union, NJ). Deer were immediately transported to the Colorado State University Veterinary Diagnostic Laboratory for a complete post mortem examination.

At necropsy of the GFD/Mte tracer control deer, the nasal cavity, throat, and head lymph nodes were examined for the presence of GFD/Mte using a hand-held UV light source (Spectroline, Westbury, NY). Nasal turbinates were serially dissected and photographed. To detect microscopic-sized dyed particles, a fluorescent microscope was used with a UV excitation source and UV emission filter.

To visualize fluorescent prions, sections were counterstained with 1 µg/mL of the Carbocyanin lipophilic tracer DiOC_18_ and 100 ng/mL DAPI for 20 minutes, then mounted using ProLong Gold fluorescent mounting medium (Life Technologies, Madison, WI). Five to twelve 5 µm thick, paraffin-embedded tissue sections from nasal turbinates, retropharyngeal, submandibular and parotid lymph nodes and tonsils were viewed and photographed using an Olympus BX60 microscope (Center Valley, PA) equipped with a cooled charge-coupled diode camera.

### Immunohistochemistry

Retropharyngeal, submandibular, parotid, pre-scapular, pre-femoral, mesenteric, and illiocecalcolic junction lymph nodes as well as tonsils, Peyer's patches, rectum, brain, and nasal tissues (cribriform plate, and ethmoid turbinates, two sections from each) were collected from the deer at necropsy and placed in 10% neutral buffered formalin for one week. The nasal tissues were then placed in 10% formic acid for three to five days for decalcification, trimmed, and embedded in paraffin blocks. Other tissues were trimmed and two to four sections of each were placed in plastic cassettes and allowed to fix for an additional two days. Slides were prepared as previously described for visualization and evaluation [27]. Briefly, 5 µm tissue slices were mounted on positively charged glass slides (Fisher Scientific, Houston, TX), antigen retrieval with formic acid and hydrated autoclaving was performed and F99/97.6.1 antibody was employed to detect PrP^CWD^, a biomarker for CWD, followed by incubation with alkaline phosphatase-conjugated anti-mouse IgG secondary antibody and visualized using an automated immnuostainer and an alkaline phosphatase red kit (Ventana, Tucson). Slides were counterstained with hematoxylin for four minutes at 37°C. Positive and negative control slides containing lymphoid tissues and brain were stained with each run. PrP^CWD^ was visualized as granular red staining showing protease-resistant deposits within lymphoid follicles. All follicles were counted for each section present on each slide, totaling between 1600–3069 follicles for each deer.

## Results

### Chronic wasting disease infection

**Figure 2:**
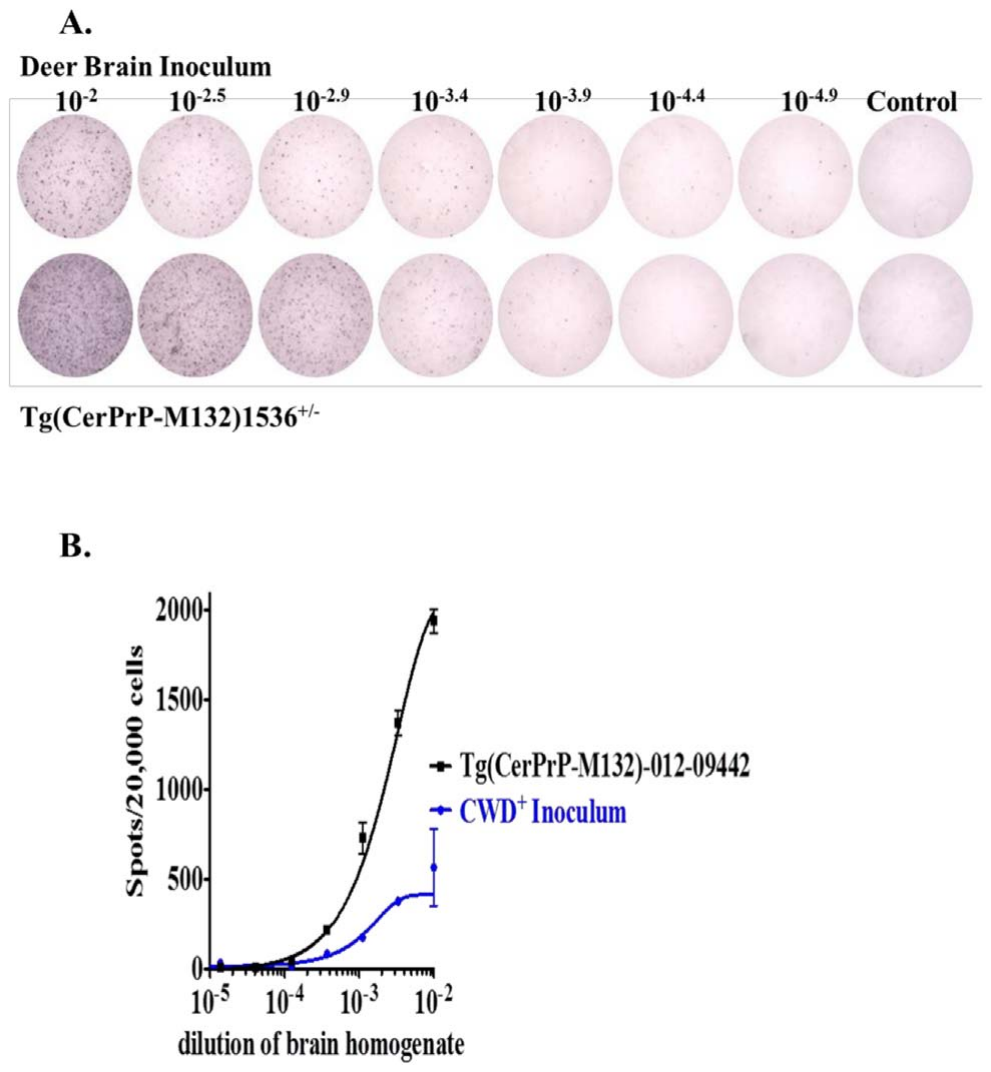
Quantification of deer CWD prion by the cervid prion cell assay. **A.** Representative wells of an ELISPOT plate showing spots given by triplicate deer 5E9-S1cells exposed to 3-fold serial dilutions of CWD+ inoculate brain homogenate, deer CWD (*upper*) and Tg(CerPrP-M132)1536^+/−^-passaged CWD prion isolate 012-09442 (lower), the concentration of brain homogenates (*left* to *right*) are: 10^−2^, 10^−2.5^, 10^−2.9^, 10^−3.4^, 10^−3.9^, 10^−4.4^ and 10^−4.9^. The wells at right are uninfected Deer5E9-S1 cells. **B.** Responsiveness of Deer5E9-S1 cells to the CWD+ inoculum and Tg(CerPrP-M132)1536^+/−^ mouse -passaged CWD prion isolate 012-09442 were between 10^−2^ and 10^−5^. The cells were infected with serial 1∶3 dilutions of homogenates of CWD+ inoculum and subjected to the CPCA. In each case, the mean is derived from experiments performed in triplicate, with error bars indicating the standard errors of the means (SEM).

The CWD status of the negative inoculum was verified by PMCA and the positive by western blot analysis (data not shown) prior to inoculation. Control brain inoculum remained negative after six 24 hr rounds of PMCA amplification and western blot visualization. Positive brain inoculum was positive on western blot visualization. The deer CWD prion infectivity titer was determined by CPCA using a highly sensitive Deer 5E9-S1 cell line (Fig. 2). The prion titers of the inoculum and a Tg(CerPrP-M132)1536^+/−^ mouse-passaged CWD prion isolate 012-09442 were calculated as 10^6.1^ and 10^6.6^ cervid prion cell units per gram of brain homogenate, (CPCA units/g), respectively. The results of the titer assay revealed that the CWD prion inoculum titer was approximately three times lower than that of a transgenic mouse strain commonly used to generate disease in a mouse bioassay (Fig. 2). All deer were healthy upon inoculation, however, shortly after inoculation an SS deer in the 175 DPI group developed a chronic GI infection that required long-term antibiotic treatment. As a result this deer was excluded from the study.

**Figure 3:**
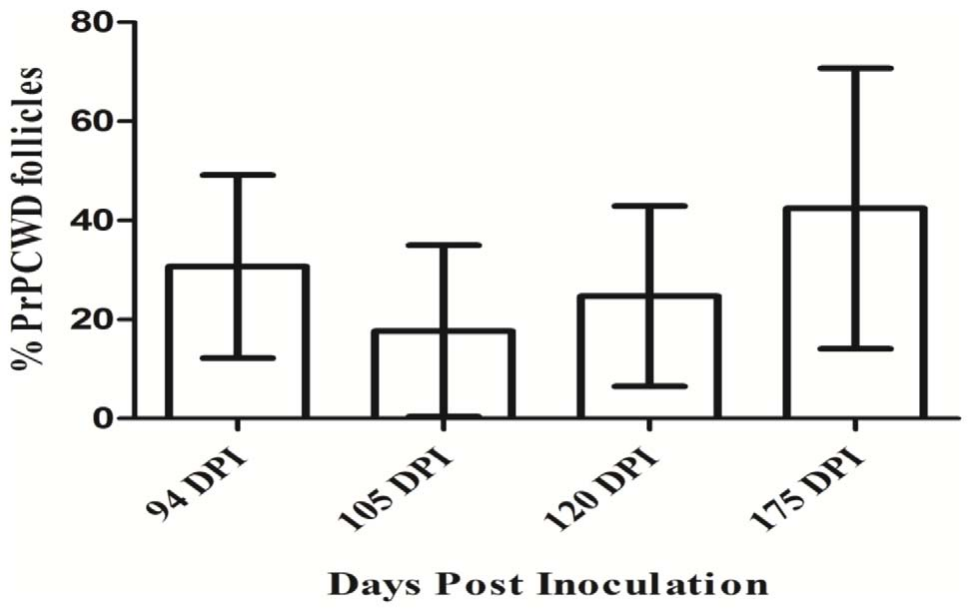
Lymphoid Involvement at each of the 4 time points. No significant difference was detected in the number of affected follicles between the groups (ANOVA, *p = *0. 86).

We observed no clinical signs characteristic of CWD and no detectable PrP^CWD^ by IHC in the obex, thalamus, hypothalamus, basal ganglia, olfactory bulb, or hippocampus of the brain in any of the deer at any of the time points. We next investigated subclinical infection by searching for PrP^CWD^ in lymph nodes that drain or are proximal to the nasal cavity; retropharyngeal, submandibular, parotid, and palatine tonsil, as well as distal lymph nodes, pre-scapular, pre-femoral, mesenteric, Peyer's patches and the recto-anal mucosa associated lymphoid tissue (RAMALT). PrP^CWD^ was not detected in either of the two mock-infected control deer (0/2 at 175 DPI), but was detected in lymph nodes of 10/11 of the CWD-inoculated deer by IHC (Table 1) as early as 94 DPI. The number of CWD-positive follicles was not significantly different at later time points (Fig. 3).

**Table 1:** Detection of CWD in lymphoid follicles.

Codon 96	DPI	Number of CWD+ follicles	% of affected
genotype		total follicles	follicles
GG	175	0/2547	0%
GS	175	0/2900	0%
GS	95	663/2141	31%
GG	95	1376/1972	70%
SS	95	66/2939	2%
GG	105	828/1635	51%
SS	105	0/2338	0%
SS	105	20/3069	<1%
GG	120	1751/2396	73%
GS	120	155/1763	9%
SS	120	168/2061	8%
GG	175	467/2790	17%
GS	175	471/1790	26%

Between 1600 and 3069 lymphoid follicles from head LNs, tonsils, pre-scapular and femoral LNs, Peyer's patches, mesenteric LN, gut LNs and rectum were evaluated in each deer for the presence of CWD by IHC. Percentage of total follicles IHC-positive for CWD ranged between 0->70%, depending on genotype. (*Control mock-infected deer).

### Influence of genotype

**Figure 4:**
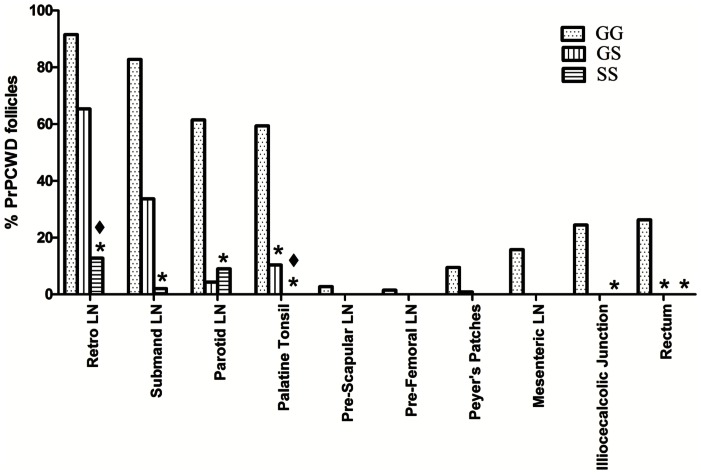
Prnp genotype affects PrP^CWD^ distribution and proportion of PrP^CWD^ positive follicles in the lympoid system. The GG genotype had a wider PrP^CWD^ distribution than GS and SS. *Tissue CWD % was significantly less than the GG genotype. ♦ Tissue CWD % was significantly less than the GS genotype. (Students one tailed T-test, *p*≤0.05).

**Figure 5:**
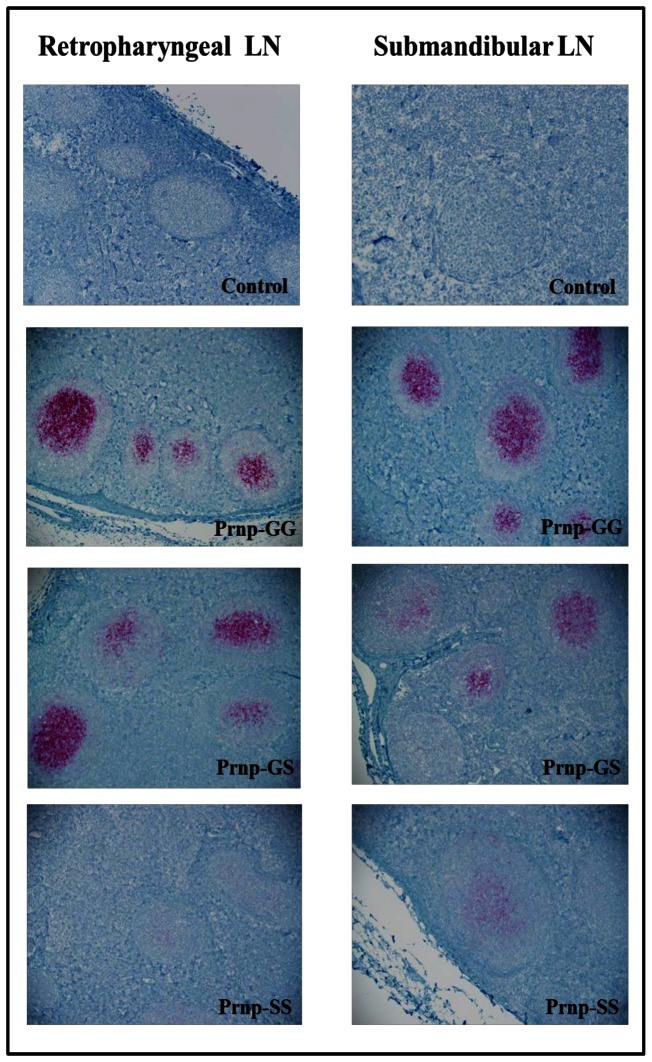
PrP^CWD^ immunolabeling in the retropharyngeal and submandibular lymph nodes at 200× magnification, mAb F99/97.6.1 and alkaline phosphatase detection.

Upon genotypic analysis, it was discovered that although all deer were homozygous at codon 95 (QQ), all three genotypes (GG, GS, and SS) were represented at codon 96. We therefore sampled deer from each genotype at each time point. We found that the genotype at codon 96 affected PrP^CWD^ temporal distribution and proportion of PrP^CWD^ positive follicles within the lymphoid system (Fig. 4). Overall, the deer with GG genotype had a much wider PrP^CWD^ distribution in the body and a higher percentage of CWD-positive follicles in the retropharyngeal, submandibular, parotid, pre-scapular, pre-femoral, and mesenteric lymph nodes, palatine tonsils, Peyer's patches, ileocecocolic junction, and rectum. The number of CWD+ immunoreactive follicles in the retropharyngeal, submandibular, parotid, palatine tonsils, illiocecalcolic junction, and rectum were significantly greater (Student's one-tailed T-test *p = *<0.05) in the GG animals as compared to SS deer. Follicle number in the palatine tonsils and the rectum were significantly greater (Student's one-tailed T-test *p = *<0.05) than in GS deer (Fig. 4). Deer having the GS genotype had fewer positive follicles and a more limited distribution with positive follicles detected in the retropharyngeal, submandibular, and parotid lymph nodes, palatine tonsils and Peyer's patches (Fig. 4). We found the most limited distribution and lowest number of positive follicles in the deer with the SS genotype, with PrP^CWD^ detected only in the retropharyngeal, submandibuliar, and parotid lymph nodes (Fig. 4). Genotype also had an influence on the intensity of immunoreactivity within the affected lymph nodes, with GG having the greatest number of stained granules, followed by GS then SS (Fig. 5).

### Inoculum tracking

**Figure 6:**
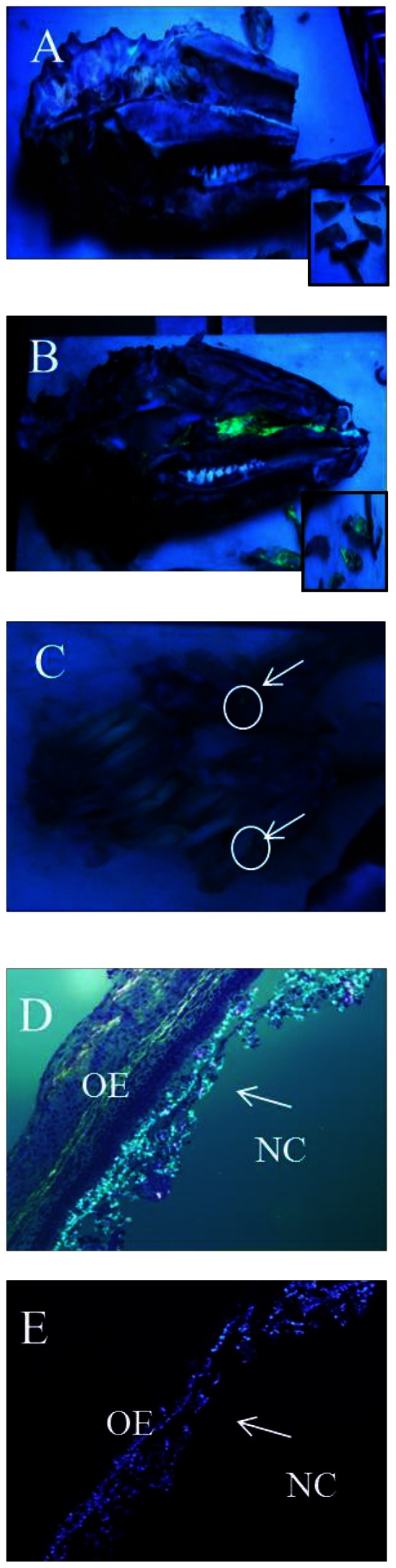
Visualization of GFD/Mte particles. 6A. Longitudinal cut of a deer head without GFD/Mte under a blacklight. Inset- dissected nasal turbinates. 6B. Longitudinal cut of a deer head inoculated with GFD/Mte under a black light with visible GFD/Mte deposition. Insets- dissected nasal turbinates 6C. Ethmoid nasal turbinates under a blacklight. Arrows and circles indicate small particles of GFD/Mte. 6D. Mounted nasal turbinate with 100× magnification showing GFD/Mte particles associated with the nasal mucosa under normal light and with a ultraviolet filter. OE-Olfactory epithelium, NC-Nasal Cavity. Arrows indicate GFD/Mte (6E).

**Figure 7:**
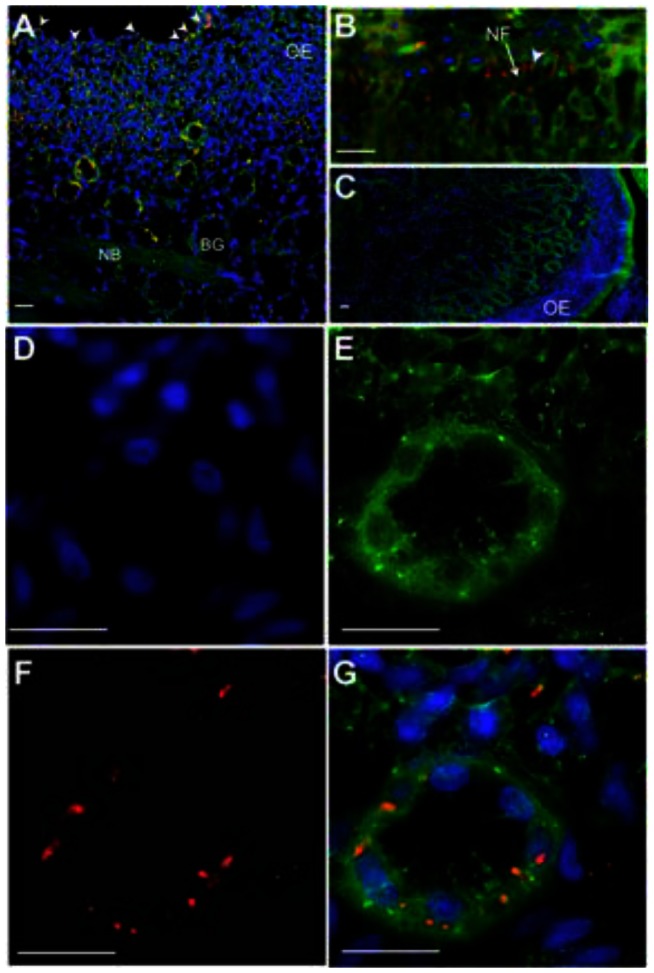
Tracking lyophilized prion inocula in the nasal cavity of deer. Highly enriched, fluorescently labeled prions were mixed with Mte, lyophilized, pulverized and puffed into the nasal cavity. (A) After 45 minutes, florescent prion aggregates (red) can be seen on (white arrowheads) and within the olfactory epithelium (OE) of the nasal turbinates. Tissue sections are counterstained with DiOC_18_ fluorescent membrane dye (green) and the nuclear stain DAPI (blue). A small proportion of prions can be seen near serous cells of the Bowman's glands (BG) in the lamina propria. (B) By 60 min, significant amount of prions were found in the lamina propria, with some aggregates (arrowhead) associated with nerve fibers (NF) emanating from the OE. (C) We detected no red signal from negative control sections from mock-inoculated deer. (D–G) Higher magnification of a Bowman's gland stained with DAPI (D), DiOC_18_ (E) and decorated with prions (F) that appear to localize on serous cells (G). NB, nerve bundle; scale bar, 20 µm.

To determine if the deposition of the initial inocula could influence later replication sites and to understand the deposition and initial contact/entry sites for prions and the Mte within the nasal cavity, we sought to track the location of inocula immediately after exposure. We first puffed GFD/Mte dust (Fig. 1) into the nasal cavity and searched for it 45 min later. The Mte is not readily visualized without the presence of the GFD. Upon gross examination, we saw a small amount of GFD/Mte under visible light and a significant amount under UV light on the nasal mucosa of the nasal passages and to a lesser extent in the nasopharanx (Fig. 6a). A few small spots were seen on the ethmoid turbinates (Fig. 6b). Microscopic examination revealed GFD/Mte on the mucus and surface epithelial cells of the nasal cavity (Fig. 6 c, d, e). We next puffed highly-enriched fluorescent prions adsorbed to Mte into the nasal cavity and searched for them microscopically based on the location of GFD/Mte. We found fluorescently tagged prion rods in both animals after 45 and 60 min on and within the olfactory epithelium (OE, Fig. 7A), as well as associated with nerve fibers (NF, Fig. 7b) and serous cells of the Bowman's glands (BG) in the lamina propria (LP) after 60 min (Fig. 7D–G).

We detected no prions associated with blood or lymphatic vessels in the lamina propria, or in any of the lymph nodes examined, including the retropharyngeal, submandibular and parotid lymph nodes, and palatine tonsils at these very early time points.

## Discussion

Indirect environmental CWD transmission has been demonstrated and several infectious tissues and excreta have been identified that could contaminate forage, soil and water [5], [6], [7] [28]. We also know that prions can adsorb to soil and its components and remain infectious [16]. However, we know little about how prions are re-introduced into hosts from the environment. Here we model natural prion exposure to dry, prion-adsorbed Mte particles that mimics natural, repeated IN dust exposure at a volume significantly lower (1.2 g) than those used in previous oral inoculation studies (5 and 10 g respectively) [14], [15]. We failed to detect PrP^CWD^ in the brain as late as 175 days after IN PrP^CWD^ dust exposure. All brain regions analyzed were negative, demonstrating that although PrP^CWD^ staining could be detected in the lymph nodes of the head in all genotypes and in more distal nodes in the GG genotype, observable PrP^CWD^ accumulation had not yet progressed to the brain.

The olfactory bulb was of particular interest in this study. There are millions of olfactory sensory neurons (OSN) present in the nasal mucosa that connect directly to the olfactory bulb [29], [30]. Diseases such as polio, rabies and herpes simplex 1 infection [31]–[33] and toxicants such as aflatoxin B [34] are able to take advantage of this direct anterograde connection to infect the brain. It has been suggested that infectious prions may utilize this same route to enter the brain, as PrP^RES^ deposition has been documented in the olfactory bulb and nasal mucosa of terminally ill, infected animals [35]. Additionally, both the OSNs and olfactory bulb express PrP^C^ in mice [30]. In this study, the olfactory bulb, surrounding brain area and nasal mucosa did not show deposition of PrP^CWD^ by IHC, demonstrating that the PrP^CWD^ did not travel along these neurons to enter the brain by 175 DPI. Our results are in agreement with rodent studies that show prion diseases are unable to make use of this direct connection to the brain and that olfactory bulb and nasal mucosa deposition occurs later in the disease, moving retrograde from the brain to the olfactory bulb and then down the OSNs to the nasal mucosa [20], [35].

In contrast to the brain, we found substantial PrP^CWD^ in the lymph nodes and tonsils of the head (retropharyngeal, submandibular and parotid lymph nodes) in all genotypes. The tonsil findings are in agreement with those of a concurrent study using aerosolization of liquid inoculum [22]. We detected PrP^CWD^ in mesenteric lymph nodes, Peyer's patches and in lymphoid follicles at the ileocecalcolic junction of the gut and rectum only in deer expressing the GG PRNP genotype. Despite detecting original inoculum deep within the nasal turbinates on OE and mucus-secreting Bowman's glands, IHC analysis of the nasal turbinates did not reveal the presence of CWD prions later in infection. Taken together, these data suggest that after IN exposure, CWD infection begins in lymphoid tissues proximal to the nasal cavity and later progresses to the brain.

Dust can be an environmentally realistic, chronic, low dose mode of intranasal CWD exposure for deer and elk in the wild, particularly in dry western states such as Colorado and Wyoming. The size of dust particles is directly proportional to how far the particles are able to travel into the respiratory tract, with particles ≤1 µm able to travel into the deepest regions of the lung, 1–5 µm into the bronchiolar region, 5 µm into the upper lung and >10 µm caught in the nose and oral pharynx [36]. Analysis of the Mte used in this study revealed that 2.5% of the particles were 10 µm, 5.3% were 5 µm, 36.4% were 2 µm and 55.8% were ≤1 µm in accord with a previous study evaluating Mte particle size [37]. Studies have shown that CWD can tightly adsorb to clay particles, such as Mte, and cause disease after oral inoculation [9]. In this study, Mte was used as an effective dust carrier to transmit CWD intranasally, however, further research needs to be conducted to determine the binding relationship between the CWD and the Mte. Mte is a widely distributed, biologically inert clay, commonly called Fuller's earth or bentonite. The large surface area, negative charge and small size make Mte an effective nanoparticle carrier for gene therapy DNA and chemotherapeutic agents into the gut [37]–[39]. The binding of Mte to DNA and drugs significantly enhances their mucoadhesion, cellular uptake and efficacy within the gut; and confocal microscopy reveals that Mte/drug particles are internalized into cells [37]–[39]. In a study on gut particle uptake, 1 µm latex beads were detected in Peyer's patches and mesenteric lymph nodes six hrs after oral inoculation [40]. Our gross examination of deer exposed to GFD/Mte tracer demonstrated the extensive mucoadhesion of GFD/Mte inoculum throughout the main nasal passage, associated nasal mucosa, and mucus on peripheral nasal turbinate surfaces 45 min after inoculation. It seems likely that the mucoadhesion increased retention time of the inoculum and may have facilitated increased CWD prion uptake. The initial experimental design of this study included inoculating dried brain as well as brain/Mte, however, drying the brain homogenate proved problematic. The texture of the dried brain was extremely thick and sticky, prohibiting the generation of a powder. As a result this component was eliminated.

We detected fluorescent prions on and within the olfactory epithelium and associated with nerve fibers and serous cells of the Bowman's glands in the lamina propria within one hr of exposure. We hypothesize that PrP^CWD^ traffics from the nasal mucosa to draining lymph nodes via immune cells, or autonomously through lymphatic drainage, or both [26].

Peyer's patches in the gut and the nasal mucosa contain dendritic cells (DC) and specialized membranous (M) cells [41]–[43], which sample the lumen for antigens and present them to regional lymph nodes [44]–[48]. Uptake of prions by DCs in the gut has been implicated in prion disease transmission [49], [50] with M cells can trafficking prions across cellular and mucosal surfaces in vitro and in vivo [51], [52]. The presence and importance of DCs in the nasal mucosa as first line of defense against inhaled microbes and allergens, is well established [53]–[56]. DCs reside throughout the olfactory epithelium at an airway density between 500 and 1000 cells/mm^3^ of nasal epithelium [57] and often sit atop, as well as insert between tight junctions of, the Bowman's glands [58]. We propose that in this study DCs and M cells likely took up PrP^CWD^ from the olfactory epithelium and lamina propria and transported them to regional draining lymph nodes. However, we cannot eliminate paracellular or cell autonomous movement of prions, as has previously been shown [26], [59]. Inhalation of dust and other particles has been shown to cause rhinitis that enhances cytokines and increases the number of DCs in the nasal mucosa [55], [60]–[62].

### Influence of genotype

This study provided a unique opportunity to observe the effect of the dimorphism at codon 96 on IN infection of deer with Mte-adsorbed prion particulate. Several studies examining the prevalence of codon 96 polymorphisms have been done on wild populations with the GG polymorphism being the most common (72%), followed by GS (13–25%), and SS (3%) [63], [64]. Wild GG animals have a higher prevalence and shorter incubation period of CWD compared to GS and SS genotypes [65]. The distribution and prevalence of CWD within the body of the SS and GS deer in this study were significantly different from GG wild-type deer, with GS and SS having CWD deposition predominantly in the head lymph nodes. Lymph nodes are essential sites of CWD propagation in deer and elk [66]. We detected minor PrP^CWD^ deposition in the Peyer's patches in one GS deer. These data suggest that GS and SS deer have slower CWD progression in the peripheral lymphoid tissues of the body, which may explain the delayed progression of CWD observed in GS and SS mule deer by Miller *et. al* [67].

The results of this study confirm that CWD can be successfully transmitted IN as a lyophilized prion particulate adsorbed to Mte and that genotype at codon 96 affects the lymphoid distribution of CWD within the body. Additionally, two novel intranasal tracking methods were employed that provided insight into CWD translocation within the nasal cavity. The data collected in this study may also shed light on why there is a higher prevalence of CWD in males, as males participate in more behaviors that generate dust. We propose chronic, long-term exposure to CWD prions adsorbed to dust particles to be a natural CWD infection route in addition to chronic oral and nasal contact exposure.
